# Introduction of inactivated poliovirus vaccine in the Philippines: Effect on health care provider and infant caregiver attitudes and practices

**DOI:** 10.1016/j.vaccine.2018.09.028

**Published:** 2018-10-24

**Authors:** Anna Lena Lopez, Jennifer B. Harris, Peter Francis Raguindin, Josephine Aldaba, Merrylle Morales, Patrick Sylim, Kathleen Wannemuehler, Aaron Wallace, Daniel C. Ehlman, Terri B. Hyde, Kimberley K. Fox, Batmunkh Nyambat, Maria Joyce Ducusin, Lee M. Hampton

**Affiliations:** a Institute of Child Health and Human Development, National Institutes of Health, University of the Philippines Manila, Philippines; b Global Immunization Division, Center for Global Health, Centers for Disease Control and Prevention, United States; c National Telehealth Center, National Institutes of Health, University of the Philippines Manila, Philippines; d World Health Organization, Regional Office for the Western Pacific, Philippines; e Family Health Office, Disease Prevention and Control Bureau, Department of Health, Manila, Philippines

**Keywords:** Multiple vaccine injections, Vaccine acceptance, Vaccine hesitancy, PHILIPPINES, IPV

## Abstract

**Background:**

The introduction of inactivated poliovirus vaccine (IPV) to the Philippines’ national immunization schedule meant the addition of a third injectable vaccine at a child’s 14-week immunization visit. Although previous studies have shown that providing multiple vaccines at the same time affected neither the risk of severe adverse events nor vaccine efficacy, concerns were raised that providing three injections at a single visit, with two injections in one leg, might be unacceptable to health care providers (HCP) and infant caregivers.

**Methods:**

We conducted pre- and post-IPV introduction surveys on the acceptance and acceptability of the additional injectable vaccine in three of the Philippines’ 17 administrative regions. Regions 3 and 6 were included in the pre-introduction phase and Regions 3, 6 and 10 were included in the post-introduction phase. Thirty public health centers (PHCs) were randomly sampled from each region. HCPs and infant caregivers were interviewed. In addition, vaccination records from a minimum of 20 eligible children pre-introduction and 10 children post-introduction per PHC were reviewed.

**Results and discussion:**

We interviewed 89 HCPs and 286 infant caregivers during the pre-introduction phase and 137 HCPs and 455 caregivers during the post-introduction phase. Among 986 vaccination records reviewed post-introduction, 84% (n = 826) of children received all three recommended injections at one visit, with a range from 61% (209/342) in Region 10 to 100% (328/328) in Region 3. The proportion of HCPs reporting that they had administered three or more injectable vaccines and the proportion of caregivers that would be comfortable with their child receiving three or more injectable vaccines at one visit increased from pre- to post-introduction (p < 0.0001 for both). Eighty-seven percent of HCPs that had administered three or more injectable vaccines post-introduction reported being comfortable or very comfortable with the number of vaccines they had administered.

## 1. Introduction

As part of the global cessation of use of the trivalent formulation of oral polio vaccine (tOPV, containing attenuated strains of types 1, 2, and 3 polioviruses) [1,2], the Philippine national immunization program switched from tOPV to the bivalent OPV formulation (bOPV, containing attenuated strains of types 1 and 3 polioviruses) in 2016 [3]. Prior to the worldwide cessation of tOPV use, the Global Polio Eradication Initiative (GPEI) recommended that countries introduce inactivated poliovirus vaccine (IPV) into the national immunization schedule to help protect children against type 2 vaccine-derived polioviruses should they circulate after tOPV use discontinuation [1,4]. Adhering to this GPEI recommendation, the Philippines introduced IPV in 2014 using a phased approach across its 17 regions, with introduction completed nationwide in 2016.

In many countries, IPV introduction increased the number of injectable vaccines administered to children at an immunization visit. Policymakers, particularly from developing countries, were concerned with safety, vaccine effectiveness, and health care provider (HCP) and parental acceptance of the increased number of injectable vaccines [5]. Prior research shows that administration of three injectable vaccines at one visit does not carry increased risks of serious adverse events, compared to a schedule giving fewer vaccines at a visit [6-8]. Most studies assessing infant caregiver and HCP experiences with multiple injections were from high income countries [9]; however, recent studies from Africa [10,11] confirm findings that HCPs and caregivers will very often accept the administration of three or more injectable vaccines [9].

Like many countries, the Philippines introduced IPV at the 14-week immunization visit, creating a schedule that has three injectable vaccines at this visit: IPV, pneumococcal conjugate vaccine (PCV), and pentavalent vaccine, which protects against diphtheria, tetanus, pertussis, hepatitis B and *Haemophilus influenzae* type b. PCV was introduced into the Philippines’ childhood immunization schedule during a similar timeframe as IPV, resulting in some regions increasing from one to three injections at the 14-week visit over a very short period of time. Our evaluation aimed to understand the perception, understanding, and acceptability of multiple injectable vaccines among the caregivers of children receiving their 14-week vaccinations and among HCPs administering the vaccines.

## 2. Methods

The Philippines has 17 administrative regions. We administered pre-IPV introduction surveys in two regions (3 and 6) and post-introduction surveys in three regions (3, 6 and 10). These regions had previously introduced PCV and were the earliest to introduce IPV. In addition, each region comes from a different major island group of the Philippines: Luzon (Region 3), Visayas (Region 6), and Mindanao (Region 10). Region 10 did not have a pre-introduction survey as IPV was already introduced at the time of study implementation. Post-introduction surveys were conducted at least two months after IPV introduction.

All three regions have urban and rural areas. Region 3 has 119 main public health centers (PHCs) across seven provinces and two highly urbanized cities that serve an estimated 11,124,400 population including 300,300 under 1 year old. Region 6 has 147 PHCs covering a population of 8,317,800 with 224,600 children under 1 year in six provinces and two highly urbanized cities. Region 10 has 122 PHCs for a population of 4,799,700 including 129,600 children under 1 in five provinces and two highly urbanized cities [12].

### 2.1. Sample size and selection of public health centers

The sample size of PHCs for each region was calculated to test whether there was a 5% or greater decrease in the proportion of eligible children receiving all recommended injectable vaccines, with the PHC being the unit of analysis, in a pre-post design. To detect a 5% difference using a one-sided Wilcoxon test with a significance level = 0.05 and a standard deviation of 10%, 29 PHCs were required to achieve 81% power; we rounded up to 30. PHCs were selected by simple random sampling from a list of all PHCs in each region. The same PHCs were included in the pre- and post-introduction surveys.

### 2.2. Health care provider and caregiver surveys

At the selected PHC, HCPs that had administered at least 10% of that PHC’s routine vaccinations in the prior three months were asked to participate as they had recent experience with the PHC’s routine immunization program. HCPs who had administered vaccinations only during immunization campaigns were ineligible. Where possible, the same HCPs were interviewed pre- and post-introduction.

A convenience sample of five infant caregivers were interviewed at each PHC. Eligible caregivers were adults aged ≥ 18 years who brought an infant aged ≥14 weeks to a selected PHC for a vaccination visit where the child was eligible to receive pentavalent vaccine and PCV during the pre-introduction phase, or pentavalent vaccine, PCV and IPV post-introduction. If five interviews were not achieved during one visit to the PHC, survey staff made repeat visits to attain the required number.

### 2.3. Immunization records review

During the pre-IPV introduction survey, we reviewed vaccination register records from birth through the 14-week visit for the five children whose caregivers were interviewed plus the 15 children that most recently attended their 14-week visit at that PHC. To ensure that stockouts did not affect the results of the post-IPV introduction survey, study staff checked that all 3 vaccines (IPV, PCV and pentavalent) were available in the PHCs when the post-introduction survey was conducted. Surveyors visited PHCs only when all three vaccines were in stock and abstracted records on all children eligible for their 14-week vaccinations on that day. To facilitate this prospective record review, the minimum number of records per PHC was reduced from 20 to 10, including the five whose caregivers were interviewed. If staff had to make return visits to achieve this target, they abstracted records from all eligible children on the date of the return visit.

### 2.4. Data management and analysis

Surveys were conducted using password-protected Android tables with pre-installed applications [13]. Survey responses and the numbers of injectable vaccines received were summarized. Pre-post comparisons included only regions 3 and 6. For the subset of HCPs interviewed in both phases, changes were evaluated using McNemar’s test. To exclude non-independent observations, one HCP was randomly selected for inclusion in pre-post analyses for one PHC that had two HCPs interviewed in both phases. Caregiver attitudes pre- and post-introduction were evaluated using Cochran-Mantel-Haenszel tests of general association, adjusting for PHC. Some of the responses have been collapsed into two categories to facilitate statistical testing.

Post-introduction vaccination records were used to stratify health facilities into those with ≥90% and <90% of children receiving all three injections at single visit. HCP attitudes in these two groups of PHCs were compared using Cochran-Mantel-Haenszel tests of general association, adjusting for region, with one randomly selected HCP per PHC (to exclude non-independent observations). A p < 0.05 was considered statistically significant. Data analysis was conducted using SAS version 9.4 (Cary, North Carolina, USA).

### 2.5. Ethical consideration

Ethical approval was obtained from the University of the Philippines Manila Research Ethics Board (UPM-REB-2015-349-01) and from the WHO Regional Office of the Western Pacific Ethical Review Committee (2015.25.PHL.5.EPI).

## 3. Results

The pre-introduction phase was conducted from October to December 2015 and the post-introduction phase from January to October 2016. During the pre-introduction phase, 92% and 84% of children in Regions 3 and 6, respectively, could not receive PCV and pentavalent vaccines simultaneously due to a pentavalent stockout at the central level. Modified procedures during the post-introduction phase ensured that stockouts did not affect the results of that phase. Since we could not assess correct administration of two vaccines pre-introduction, we were unable to estimate the change in receipt of all recommended injectable vaccines across phases. However we were still able to assess the correct receipt of all three vaccines post-introduction as well as the perception, understanding and acceptability of multiple vaccine injections among caregivers and HCPs during both phases.

Post-introduction, we reviewed vaccination records of 986 children (465 children of interviewed caregivers and an additional 521 children identified through vaccination registers). Of these, 826 (84%) received all three injections at one visit, with substantial variation among regions. While 100% of children in Region 3 received all three injections, only 91% in Region 6 and 61% in Region 10 received three injections at the same visit (Table 1).

Looking at administration of multiple injections within PHCs, all 30 PHCs in Region 3 had 100% of children receive three injections at the same visit. Twenty-five PHCs (83%) in Region 6 and 17 PHCs (57%) in Region 10 administered three vaccines to all children assessed. Seven PHCs (23%) in Region 10 did not administer all three injections to any children assessed (Table 1).

### 3.1. Health care providers

We interviewed 89 HCPs pre-introduction and 137 HCPs post-introduction, the majority of whom were midwives. Thirty-four (38%) of HCPs had already administered ≥3 injectable vaccines at one visit pre-introduction, and this increased to 92% (n = 126), post-introduction. Eighty-seven percent (n = 110) of those that had administered ≥3 injections post-introduction were comfortable or very comfortable giving that number (data shown stratified by region, Table 2).

When asked how many injectable vaccines they were willing to administer at one visit, 65% (n = 58) of HCPs said pre-introduction that they were willing to administer ≥3 or any number recommended by the EPI program, while 80% (n = 109) post-introduction were willing to administer ≥3 injections (Table 2). Of 28 HCPs who stated post-introduction that they would administer only 1 or 2 injections, 26 (93%) reported that they had administered ≥3 injections. Furthermore, of 11 HCPs who had administered only two injections post-introduction, 9 (82%) said they were willing to administer ≥3 injections. Among 109 HCPs who were willing to administer ≥3 injections in one visit post-introduction, the top reasons given were to provide maximum protection against disease (72%, n = 78) and to follow the vaccination calendar (71%, n = 77). The top reason cited among 28 HCPs willing to give a maximum of two injections post-introduction was to avoid too much pain and discomfort for the child (64%, n = 18) (data not shown).

Fifty (36%) HCPs interviewed post-introduction had experienced problems with caregiver acceptance of two or three injectable vaccines at one visit; Region 10 had the highest proportion of HCPs reporting problems (47%, n = 24). Nearly all HCPs (98%; n = 84) in regions 3 and 6 perceived post-introduction that all or most parents in their community would allow their children to receive three injections in one visit, while only 76% (n = 39) of HCPs in Region 10 had the same sentiment. The vast majority of HCPs in all regions and phases agreed that it was better for a child to receive more injectable vaccines in one visit if it resulted in better protected against diseases and that it was better for children to receive three injections in one visit than spread them out over three visits. A minority of HCPs (30% pre-introduction; 32% post-introduction) felt that there would be fewer side effects from two injections at separate visits rather than the same visit (Table 2).

### 3.2. Caregivers

We interviewed 286 caregivers pre-introduction and 465 caregivers post-introduction (Table 3). Most caregivers (81% pre-introduction and 86% post-introduction) were mothers of the infant. Post-introduction, 65% (n = 300) of caregivers reported being comfortable with their child receiving ≥3 injections or any number recommended by the HCP and 93% (n = 279) of these caregivers’ infants received all three vaccines. While 35% (n = 165) of caregivers post-introduction reported comfort with only one or two injectable vaccines, 68% (n = 112) of these infants received three injections (data not shown).

Caregivers whose children received <3 injections post-introduction were asked why they did not receive all three vaccines; multiple responses were permitted. Data were available from all 13 caregivers in Region 6 but only 33 of 61 in Region 10 due to a surveying error. In Region 6, 46% (n = 6) of caregivers reported that the HCP did not recommend all three vaccines; 46% (n = 6) were concerned about pain or side effects; and 31% (n = 4) felt that it was too many vaccinations. In Region 10, 73% (n = 24) of caregivers reported that their infants were not offered three vaccines, 21% (n = 7) were concerned about pain or side effects; 15% (n = 5) felt that it was too many vaccinations; and 6% (n = 2) refused multiple vaccines because their child was sick (data not shown).

Among 165 caregivers who were comfortable with ≤2 vaccines post-introduction, the top reason cited was fear of adverse events (61%, n = 100). Among 300 caregivers who were comfortable with ≥3 vaccines or any number recommended by the HCP, the most cited reasons were trust in the HCP or the immunization program (54%, n = 161) and better protection against diseases (50%, n = 151) (data not shown). An overwhelming majority of caregivers in all regions and both phases believed that HCPs could be trusted with the number of vaccines that children should receive and that following the vaccination schedule is good for the children (Table 3).

### 3.3. Pre- and post-introduction comparisons for Regions 3 and 6

Thirty-three HCPs in Regions 3 and 6 were interviewed both pre- and post-introduction. There was an increase in the proportion of HCPs that had administered ≥3 injections (p< 0.001) (Table 4). Of 12 HCPs responding pre-introduction that they were willing to administer only one or two injections, 11 (92%) administered ≥3 injections post-introduction. The proportion of HCPs that believed all or most parents would accept ≥3 injections also increased from 82% pre-introduction to 97% post-introduction (p = 0.025).

Different caregivers were interviewed pre- (n = 286) and post-introduction (n = 308) in regions 3 and 6; all were included in the pre-post comparisons (Table 4). The proportion stating they were comfortable with only one or two injections declined from 58% (n= 166) pre-introduction to 29% (n = 88) post-introduction (p < 0.001). The proportion of caregivers who preferred children to receive more injections in one visit if it would better protect them against diseases increased from 58% pre-introduction to 85% post-introduction (p < 0.001) and the proportion that preferred to visit the PHC once for three injections rather than three times for one injection each increased from 49% pre-introduction to 77% post-introduction (p < 0.001). The proportion of caregivers that believed side effects would be greater if more vaccines were injected in a single visit decreased from 57% pre-introduction to 45% post-introduction (p = 0.014) and the proportion that thought vaccines would not work as well if many were injected in a single visit decreased from 33% pre-introduction to 21% post-introduction (p = 0.001).

### 3.4. HCP characteristics and attitudes at PHCs with high or low proportion of children receiving three injections at one visit

A post-hoc stratification grouped PHCs into those where ≥90% (n = 73 PHCs) and <90% (n = 17 PHCs) of infants received all three vaccines at one visit post-introduction (Table 5). When these two groups of HCPs were compared using one randomly selected HCP per PHC, the only significant difference was in their perception of parental acceptance of multiple injections. HCPs from PHCs where ≥90% of children received three injections were more likely to believe that all or most parents would accept three injections at one visit (p < 0.001) (Table 5).

## 4. Discussion

Studies on the acceptability of multiple injections are important to immunization programs due to the ever increasing number of vaccines incorporated into immunization schedules. However, relevant studies from developing countries are lacking [9]. Our results from the Philippines, a populous lower-middle income country in Asia, were comparable to recently published assessments in other low and middle income countries [10,14,15] We observed HCPs and caregivers to be receptive to multiple injections, with acceptance increasing after HCPs and caregivers had experience with this practice. Furthermore, our study showed that attitudes do not always align with practices; for example, some caregivers stated that they were comfortable with their child receiving only 1–2 injections at a visit yet allowed their child to receive three. Similarly, some HCPs stated that they would be willing to administer only 1–2 injections to a child at a visit, yet they administered three injections.

Practices varied across regions and PHCs. Although the study was not designed to compare regions, we found that 100% of children received all three injectable vaccines post-introduction in Region 3, while only 61% did in Region 10, despite all three vaccines being available at the PHC at the time of data collection. Stratifying by PHC in Region 10, all children received three vaccines in 17 (57%) PHCs, and none of the children did in 7 (23%) PHCs, even though all three vaccines were available at the PHC. This suggests that HCPs had a significant impact on compliance with national recommendations, a finding that was recently reported from Albania [15] and Tanzania [16]. Further supporting this theory are caregiver responses; among those in Region 10 whose children did not receive all three vaccines, the majority (73%) stated that they were not offered all three. Anecdotally, we were told at some PHCs that they deliberately spread out the scheduled vaccines over multiple visits to avoid administering 3 injectable vaccines at one visit. While differences at the PHC level suggest HCP influence, regional differences suggest that training and enforcement of national guidelines may differ among regions.

A factor in HCP’s reluctance to administer three vaccines is the overestimation of parental concerns, as was seen in a previous study [17]. In our study, in PHCs where <90% of children received all three vaccines, none of the HCPs believed that all caregivers would accept multiple injections. However, among caregivers whose children did not receive all three injections, the majority stated that they were not offered all three. It is likely they would have accepted the three injections if offered, as overall acceptance was very high.

While the pre-post design with data from both HCPs and caregivers provided rich information, there were several limitations. First, nationwide stock-outs of pentavalent vaccine precluded us from assessing receipt of all recommended injectable vaccines pre-IPV introduction and hence determining whether there was a change post-introduction. Second, we were unable to conduct the pre-introduction survey in Region 10. As this Region had the lowest compliance with multiple injections post-introduction, it would be interesting to know baseline attitudes and practices there. Third, we had to replace two PHCs each in Regions 3 and 10, and six in Region 6, due to a lack of immunization activities and/or security issues and this may affect the generalizability of the findings. Fourth, a survey error occurred resulting in loss of pre-introduction HCP data from 2 PHCs and caregiver data from 1 PHC. This data loss may have occurred during transmission as some areas had spotty internet connection. Subsequently, to ensure no data loss, information was stored in the tablet with paper forms completed as a back-up. Lastly, since we chose one region from each major island group, survey questions and statements had to be translated into 3 languages (Tagalog, Bisaya and Hiligaynon) which may have affected interpretation. However, surveyors were local staff that were facile in local languages. During trainings, each question was reviewed by the local team to ensure standardized translation.

Our study documented perceptions and experiences on the introduction of a new injectable vaccine into an increasingly busy immunization schedule. The information generated from this study was used to design training and educational packages for HCPs and the general public in the Philippines. Administering multiple vaccines in a single visit provides protection to children at the earliest possible age and minimizes the impact of additional vaccines on already-limited resources for delivering them. Contrary to concerns raised by policymakers regarding IPV introduction [16], there is general acceptance of multiple injections by both HCPs and caregivers in the Philippines.

## Figures and Tables

**Table 1:** Concurrent injections at child and health facility level in three regions in the Philippines, post-IPV introduction survey, January – October 2016.

Outcome	Region 3^a^ n (%)	Region 6^a^ n (%)	Region 10^a^ n (%)
*Individual-level results*			
Number of injections received at one visit	n = 328 children	n = 316 children	n = 342 children
Received 3 injections	328 (100)	289 (91)	209 (61)
Received 2 injections	0	18 (6)	131 (38)
Received 1 injection	0	9 (3)	2 (1)
*PHC-level results*			
Proportion of children at the PHC receiving all 3 injections at one visit	n = 30 PHCs	n = 30 PHCs	n = 30 PHCs
All children received 3 injections	30 (100)	25 (83)	17 (57)
90–99% of children received 3 injections	0	1 (3)	0
50–89% of children received 3 injections	0	2 (7)	1 (3)
1–49% of children received 3 injections	0	1 (3)	5 (17)
None of the children received 3 injections	0	1 (3)	7 (23)

PHC = Primary Health Center.

a The Philippines is divided into 17 administrative regions.

**Table 2:** Characteristics, attitudes and practices of health care providers (HCPs) participating in pre- and post-IPV introduction surveys in three regions in the Philippines, 2015–2016.

	Pre-Introduction, n (%)		Post-Introduction, n (%)		
	Region 3^a^	Region 6^a^	Region 3^a^	Region 6^a^	Region 10^a^
	n = 49 from 28 PHCs	n = 40 from 28 PHCs	n = 31 from 30 PHCs	n = 55 from 30 PHCs	n = 51 from 30 PHCs
Mean number of HCPs surveyed per PHC	1.75	1.4	1.2	1.8	1.7
Type of HCP					
Midwife	38 (78)	36 (90)	27 (87)	30 (55)	25 (49)
Public Health Nurse	6 (12)	2 (5)	1 (3)	8 (15)	10 (20)
Contract nurses from Nurses Deployment Program	1 (2)	0	3 (10)	17 (31)	16 (31)
Community Health Nurse	4 (8)	2 (5)	0	0	0
Age Group (years)					
18–24	3 (6)	0	1 (3)	4 (7)	1 (2)
25–34	9 (18)	1 (2)	4 (13)	15 (27)	21 (41)
35–44	15 (31)	14 (35)	14 (45)	13 (24)	10 (20)
45–54	11 (22)	9 (23)	7 (23)	8 (15)	4 (8)
55 and above	11 (22)	16 (40)	5 (16)	15 (27)	15 (29)
Years as a vaccinator					
5 or fewer	15 (31)	5 (13)	7 (23)	19 (35)	25 (49)
6–10	8 (16)	1 (2)	5 (16)	4 (7)	3 (6)
More than 10	26 (53)	34 (85)	19 (61)	32 (58)	23 (45)
Average number of injections HCP administered in a vaccination visit (selfreport), median (IQR)	24 (15–30)	23 (15 –39)	30 (12 –50)	20 (5–30)	30 (20–50)
Highest number of injections given to one child during a vaccination visit					
1	3 (6)	9 (22)	0	0	0
2	23 (47)	20 (50)	0	5 (9)	6 (12)
3	22 (45)	11 (28)	26 (83)	37 (67)	35 (69)
4	0	0	5 (16)	13 (24)	9 (18)
5 or more	1 (2)	0	0	0	1 (2)
Comfort level with the highest number of injections administered to a child at one visit *(HCPs sub-grouped by answer to previous question)*					
1 injection	n = 3	n = 9	n = 0	n = 0	n = 0
Very comfortable	3 (1 0 0)	6 (67)	-	-	-
Comfortable	0	3 (33)	-	-	-
Not comfortable	0	0	-	-	-
Very uncomfortable	0	0	-	-	-
2 injections	n = 23	n = 20	n = 0	n = 5	n = 6
Very comfortable	7 (30)	13 (65)	-	2 (40)	2 (33)
Comfortable	14 (61)	6 (30)	-	3 (60)	4 (67)
Not comfortable	2 (9)	1 (5)	-	0	0
Very uncomfortable	0	0	-	0	0
3 injections	n = 22	n = 11	n = 26	n = 37	n = 35
Very comfortable	3 (14)	5 (45)	11 (42)	10 (27)	13 (37)
Comfortable	16 (73)	5 (45)	13 (50)	23 (62)	15 (43)
Not comfortable	3 (14)	1 (10)	1 (4)	4 (11)	7 (20)
Very uncomfortable	0	0	1 (4)	0	0
4–5 injections	n = 1	n = 0	n = 5	n = 13	n = 10
Very comfortable	0	-	2 (40)	5 (38)	2 (20)
Comfortable	1 (1 0 0)	-	2 (40)	8 (62)	6 (60)
Not comfortable	0	-	1 (20)	0	2 (20)
Very uncomfortable	0	-	0	0	0
Highest number of injections HCP would be willing to administer to a child at one visit					
1	2 (4)	7 (17)	0	2 (4)	3 (6)
2	9 (18)	13 (33)	5 (16)	10 (18)	8 (16)
3	19 (39)	12 (30)	7 (23)	16 (29)	22 (43)
4	0	1 (3)	1 (3)	10 (18)	3 (6)
5	0	0	1 (3)	4 (7)	0
Any number recommended by the national immunization program	19 (39)	7 (17)	17 (55)	13 (24)	15 (29)
Have experienced problems with caregivers’ acceptance of 2 or 3 injectable vaccines for their child at one visit?					
Yes	22 (45)	10 (25)	7 (23)	19 (35)	24 (47)
Perception of the proportion of parents in the community that would allow their children to receive 3 vaccine injections at one visit					
All	16 (33)	11 (27)	19 (61)	27 (49)	11 (21)
Most	25 (51)	17 (43)	12 (39)	26 (47)	28 (55)
Some	8 (16)	12 (30)	0	2 (4)	10 (20)
None	0	0	0	0	2 (4)
**HCPs that ‘agree’ with the following statements:**					
It is better for a child to receive more injectable vaccines at a single visit if it means that they will be better protected against diseases	46 (94)	33 (83)	31 (100)	55 (100)	48 (94)
It is better for a child to receive 3 injectable vaccinations in 1 visit rather than 1 injectable vaccination in 3 separate visits	46 (94)	29 (73)	29 (94)	51 (93)	45 (88)
There will be fewer side effects if a child receives one injectable vaccination in each of two separate visits rather than two injections in a single visit	8 (16)	19 (48)	7 (23)	16 (29)	21 (41)

PHC = Primary Health Center; IQR = inter-quartile range.

a The Philippines is divided into 17 administrative regions.

**Table 3:** Characteristics, attitudes and practices of caregivers participating in pre- and post-IPV introduction surveys in three regions in the Philippines, 2015–2016.

	Pre-introduction, n (%)		Post-introduction, n (%)		
Characteristic	Region 3^a^	Region 6^a^	Region 3^a^	Region 6^a^	Region 10^a^
	n = 140 from 29 PHCs	n = 146 from 29 PHCs	n = 157 from 30 PHCs	n = 151 from 30 PHCs	n = 157 from 30 PHCs
Mean number of caregivers surveyed per PHC	4.8	5.0	5.2	5.0	5.2
Relationship of caregiver to child					
Mother	119 (85)	113 (77)	129 (82)	126 (83)	146 (93)
Father	5 (4)	17 (12)	2 (1)	3 (2)	4 (3)
Grandmother	9 (6)	8 (6)	15 (10)	10 (7)	4 (3)
Aunt	5 (4)	5 (3)	11 (7)	8 (5)	3 (2)
Other	2 (1)	3 (2)	0	4 (3)	0
Total number of children in child’s household					
1–2	68 (49)	92 (63)	88 (56)	94 (62)	82 (52)
3–4	48 (35)	41 (28)	55 (35)	43 (28)	47 (30)
5 or more	23 (16)	13 (9)	14 (9)	14 (9)	28 (18)
Education level of caregiver					
Grade 6 or less	10 (7)	17 (12)	15 (10)	9 (6)	11 (7)
Some high school	21 (15)	21 (14)	27 (17)	19 (13)	33 (21)
High school graduate	57 (41)	50 (34)	69 (44)	55 (36)	44 (28)
Some college/technical course	31 (22)	30 (21)	28 (18)	33 (22)	38 (24)
College graduate	21 (15)	28 (19)	18 (11)	35 (23)	31 (20)
Age of caregiver in years, median (IQR)	26 (22 – 32)	28 (23 – 33)	28 (23 – 34)	28 (24 – 33)	26 (23 – 32)
Maximum number of injections caregiver is comfortable with their child receiving at one vaccination visit					
1	22 (16)	52 (36)	8 (5)	8 (5)	19 (12)
2	36 (26)	56 (38)	32 (20)	40 (26)	58 (37)
3	17 (12)	12 (8)	51 (32)	47 (31)	30 (19)
4	2 (1)	1 (1)	1 (1)	0	2 (1)
5	1 (1)	3 (2)	0	3 (2)	2 (1)
Comfortable with any number or whatever number recommended by the HCP	62 (44)	22 (15)	65 (41)	53 (35)	46 (29)
**Caregivers that ‘agree’ with the following statements:**					
The vaccinator can be trusted concerning how many vaccines your child needs to receive in a single visit	136 (97)	139 (95)	154 (98)	150 (99)	152 (97)
Following the vaccination schedule is good for your children	138 (99)	145 (99)	157 (100)	151 (100)	157 (100)
It is better for a child to receive more injectable vaccines at a single visit if it means that they will be better protected against diseases	83 (59)	74 (51)	124 (79)	128 (85)	131 (83)
There will be fewer side effects if a child receives one injectable vaccinations in each of two or three separate visits rather than two or three injections in a single visit	58 (41)	80 (55)	52 (33)	60 (40)	97 (62)
Instead of visiting the clinic on 3 occasions to provide your child with 1 injection at each visit, you would prefer to visit the clinic only once so that your child receives all 3 vaccine injections at one visit	77 (55)	60 (41)	107 (68)	117 (77)	97 (62)
You are more concerned about your child having pain and discomfort from vaccinations spread out over multiple visits than about pain and discomfort from vaccinations given all at once during a single visit	71 (51)	103 (71)	96 (61)	99 (66)	126 (80)
Vaccines will not work as well if many are injected at a single visit	30 (21)	40 (27)	26 (17)	23 (15)	44 (28)

PHC = Primary Health Center; IQR = inter-quartile range.

a The Philippines is divided into 17 administrative regions.

**Table 4:** Comparison of health care provider (HCP) and caregiver attitudes and practices in pre- and post- IPV introduction surveys in two regions of the Philippines, 2015–2016.

	Pre-introduction n (%)	Post-introduction n (%)	p-value
**Health care providers**^a^	n = 33^b^		
Highest number of injections given to one child during a vaccination visit			
1–2	19 (58)	1 (3)	<0.0001
3 or more	14 (42)	32 (97)	
Comfort level with the highest number of injections administered to one child at a single visit Comfortable or very comfortable	30 (91)	30 (91)	1.0
Uncomfortable or very uncomfortable	3 (9)	3 (9)	
Highest number of injections you would be willing to administer to one child at a single visit			
1–2	12 (36)	8 (24)	0.157
3 or more or any number recommended by the national immunization program	21 (63)	25 (76)	
Perception of the proportion of parents in the community that would allow their children to receive 3 injections at one visit			
All or most	27 (82)	32 (97)	0.025
Some	6 (18)	1 (3)	
It is better for a child to receive more injectable vaccines at a single visit if it means that they will be better protected against diseases (n = 32)			
Agree	28 (88)	32 (1 0 0)	<0.0001
Disagree	4 (12)	0	
It is better for a child to receive 3 injectable vaccinations in 1 visit rather than 1 injectable vaccination in 3 separate visits (n = 32)			
Agree	26 (81)	30 (94)	0.103
Disagree	6 (19)	2 (6)	
There will be fewer side effects if a child receives one injectable vaccination in each of two separate visits rather than two injections in a single visit (n = 32)			
Agree	7 (22)	7 (22)	1.0
Disagree	25 (78)	25 (78)	
**Caregivers**^c^	n = 286^d^	n = 308^d^	
Highest number of injections you would be comfortable with your child receiving			
1–2	166 (58)	88 (29)	<0.0001
3 or more or any number recommended by the EPI program	120 (42)	220 (71)	
It is better for a child to receive more injectable vaccines at a single visit if it means that they will be better protected against diseases (n = 566)^e^			
Agree	157 (58)	252 (85)	<0.0001
Disagree	114(42)	43 (15)	
There will be fewer side effects if a child receives one injectable vaccinations in each of two or three separate visits rather than two or three injections in a single visit (n = 492)^e^			
Agree	138(57)	112 (45)	0.014
Disagree	104 (43)	138 (55)	
Instead of visiting the clinic on 3 occasions to provide your child with 1 injection at each visit, you would prefer to visit the clinic only once so that your child receives all 3 vaccine injections at one visit (n = 569)^e^			
Agree	137 (49)	224 (77)	<0.0001
Disagree	142 (51)	66 (23)	
You are more concerned about your child having pain and discomfort from vaccinations spread out over multiple visits than about pain and discomfort from vaccinations given all at once during a single visit (n = 556)^e^			
Agree	174 (64)	195 (69)	
Disagree	100 (36)	87 (31)	0.349
Vaccines will not work as well if many are injected at a single visit (n = 452)^e^			
Agree	70 (33)	49 (21)	
Disagree	145 (67)	188 (79)	0.001

Pre-introduction: Region 3, n = 140; Region 6, n = 146.

Post-introduction: Region 3, n = 157; Region 6, n = 151.

a Comparison of paired sample using McNemar’s test from subset of 33 HCPs (maximum 1 per primary health center, PHC) in regions 3 & 6 that were surveyed both pre- and post-introduction.

b Number of HCPs from each region: Region 3, n = 14; Region 6, n = 19.

c Number of caregivers from each region: Pre-introduction – Region 3 n = 140, Region 6 n = 146 Post-introduction Region 3 n = 157 Region 6 n = 151.

d Comparison of independent samples in regions 3 and 6 pre- and post-IPV using Cochran-Mantel-Haenszel test of general association adjusted for PHC.

e Caregivers that responded “I don’t know” were excluded from this analysis; hence the number of caregivers was less than the total sample of 594.

**Table 5:** Characteristics and attitudes of healthcare providers at public health clinics (PHC), by proportion of children receiving three vaccines at one visit post-IPV introduction, the Philippines, January – March 2016.

	Health care providers from PHCs with >=90% of children receiving 3 injections (n = 109 from 73 PHCs)	Health care providers from PHCs with <90% of children receiving 3 injections (n = 28 from 17 PHCs)
Number of injections that health care provider is willing to administer to a child at one visit		
1–2	19 (17)	9 (32)
3	32 (29)	13 (46)
4, 5 or any recommended by EPI program	58 (53)	6 (21)
Age group		
Less than 35	39 (36)	7 (25)
35–54	47 (43)	9 (32)
55 and over	23 (21)	12 (43)
Years of service as a vaccinator		
5 or fewer	42 (39)	9 (32)
6–10	11 (10)	1 (4)
More than 10	56 (51)	18 (64)
Perception of the proportion of parents in the community that would allow their children to receive 3 injections at one visit		
All	57 (52)	0
Most	49 (45)	17 (61)
Some	3 (3)	9 (32)
None	0	2 (7)
**HCPs that ‘agree’ with the following statements:**		
It is better for a child to receive more injectable vaccines at a single visit if it means that they will be better protected against diseases (n= 136)	108 (99)	26 (96)
It is better for a child to receive 3 injectable vaccinations in 1 visit rather than 1 injectable vaccination in 3 separate visits (n = 135)	101 (93)	24 (89)
There will be fewer side effects if a child receives one injectable vaccination in each of two separate visits rather than two injections in a single visit (n = 135)	33 (31)	11 (41)
